# Economic evaluation of short treatment for multidrug-resistant tuberculosis, Ethiopia and South Africa: the STREAM trial

**DOI:** 10.2471/BLT.19.243584

**Published:** 2020-02-25

**Authors:** Jason J Madan, Laura Rosu, Mamo Girma Tefera, Craig van Rensburg, Denise Evans, Ivor Langley, Ewan M Tomeny, Andrew Nunn, Patrick PJ Phillips, I D Rusen, S Bertel Squire, for the STREAM study health economic evaluation collaborators

**Affiliations:** a Warwick Medical School, University of Warwick, Coventry, England.; b Centre for Applied Health Research and Delivery, Liverpool School of Tropical Medicine, Pembroke Place, Liverpool, L3 5QA, England.; c Department of Business Management, Addis Ababa Science & Technology University, Addis Ababa, Ethiopia.; d Health Economics and Epidemiology Research Office, University of Witwatersrand, Johannesburg, South Africa.; e Medical Research Council Clinical Trials Unit at University College London, Institute of Clinical Trials & Methodology, London, England.; f Department of Medicine, University of California San Francisco, San Francisco, United States of America (USA).; g Division of Research and Development, Vital Strategies, New York, USA.

## Abstract

**Objective:**

To investigate cost changes for health systems and participants, resulting from switching to short treatment regimens for multidrug-resistant (MDR) tuberculosis.

**Methods:**

We compared the costs to health systems and participants of long (20 to 22 months) and short (9 to 11 months) MDR tuberculosis regimens in Ethiopia and South Africa. Cost data were collected from participants in the STREAM phase-III randomized controlled trial and we estimated health-system costs using bottom-up and top-down approaches. A cost–effectiveness analysis was performed by calculating the incremental cost per unfavourable outcome avoided.

**Findings:**

Health-care costs per participant in South Africa were 8340.7 United States dollars (US$) with the long and US$ 6618.0 with the short regimen; in Ethiopia, they were US$ 6096.6 and US$ 4552.3, respectively. The largest component of the saving was medication costs in South Africa (67%; US$ 1157.0 of total US$ 1722.8) and social support costs in Ethiopia (35%, US$ 545.2 of total US$ 1544.3). In Ethiopia, trial participants on the short regimen reported lower expenditure for supplementary food (mean reduction per participant: US$ 225.5) and increased working hours (i.e. 667 additional hours over 132 weeks). The probability that the short regimen was cost–effective was greater than 95% when the value placed on avoiding an unfavourable outcome was less than US$ 19 000 in Ethiopia and less than US$ 14 500 in South Africa.

**Conclusion:**

The short MDR tuberculosis treatment regimen was associated with a substantial reduction in health-system costs and a lower financial burden for participants.

## Résumé

**Objectif:**

Étudier les variations de coût liées à l'adoption d'un traitement court de la tuberculose multirésistante (MR) pour les systèmes de santé et les participants.

**Méthodes:**

Nous avons comparé les coûts pris en charge par les systèmes de santé et les participants pour des schémas thérapeutiques longs (20 à 22 mois) et courts (9 à 11 mois) en Éthiopie et en Afrique du Sud. Les données ont été récoltées auprès des participants à la phase III de l'essai clinique randomisé STREAM, et nous avons estimé les dépenses assumées par les systèmes de santé en utilisant des approches ascendantes et descendantes. Enfin, pour analyser l'efficacité des coûts, nous avons calculé les frais additionnels qu'entraîne chaque issue défavorable évitée.

**Résultats:**

Les dépenses en soins de santé par participant en Afrique du Sud s'élevaient à 8340,7 dollars américains (US$) avec le traitement long et à 6618,0 US$ avec le traitement court; en Éthiopie, le montant équivalait respectivement à 6096,6 US$ et 4552,3 US$. La principale composante économique en Afrique du Sud était le coût des médicaments (67%, 1157,0 US$ sur un total de 1722,8 US$) tandis qu'en Éthiopie, il s'agissait de l'aide sociale (35%, 545,2 US$, sur un total de 1544,3 US$). En Éthiopie, les participants à l'essai clinique pour le traitement court ont signalé une baisse des dépenses consacrées à l'alimentation complémentaire (réduction moyenne par participant : 225,5 US$) et une hausse des heures de travail (c'est-à-dire 667 heures en plus sur 132 semaines). La probabilité que le traitement court soit plus rentable dépassait les 95% lorsque la valeur accordée aux issues défavorables évitées était inférieure à 19 000 US$ en Éthiopie, et à 14 500 US$ en Afrique du Sud.

**Conclusion:**

Le traitement court de la tuberculose MR a entraîné une importante diminution des dépenses pour les systèmes de santé, ainsi qu'une moindre charge financière pour les participants.

## Resumen

**Objetivo:**

Investigar los cambios en los costos para los sistemas sanitarios y los participantes, derivados del cambio a planes de tratamiento a corto plazo para la tuberculosis multirresistente (MDR, por sus siglas en inglés).

**Métodos:**

Se compararon los costos para los sistemas sanitarios y los participantes de los planes de tratamiento a largo (20 a 22 meses) y a corto plazo (9 a 11 meses) de la tuberculosis en Etiopía y Sudáfrica. Se recopilaron datos sobre los costos de los participantes en el ensayo STREAM fase III, controlado y aleatorizado y se estimaron los costos del sistema sanitario utilizando enfoques ascendentes y descendentes. Se realizó un análisis costo-efectividad calculando el costo incremental por cada resultado negativo que se evitó.

**Resultados:**

Los costos de atención sanitaria por participante en Sudáfrica fueron de 8340,7 dólares estadounidenses (USD) con el plan largo y de 6618,0 USD con el plan corto; en Etiopía, fueron de 6096,6 y 4552,3 USD, respectivamente. El mayor factor de ahorro fue el costo de los medicamentos en Sudáfrica (67 %; 1157,0 USD del total de 1722,8 USD) y los costos de apoyo social en Etiopía (35 %; 545,2 USD del total de 1544,3 USD). En Etiopía, los participantes del ensayo que siguieron el plan corto notificaron un menor gasto en alimentos suplementarios (reducción media por participante: 225,5 USD) y un aumento en las horas de trabajo (es decir, 667 horas adicionales en 132 semanas). La probabilidad de que el plan corto fuera rentable era superior al 95 % cuando el valor asignado para evitar un resultado negativo era inferior a 19 000 USD en Etiopía y a 14 500 USD en Sudáfrica.

**Conclusión:**

El plan de tratamiento a corto plazo de la tuberculosis MDR se asoció con una reducción sustancial de los costos del sistema sanitario y con una menor carga financiera para los participantes.

## ملخص

الغرض استقصاء التغيرات في تكلفة النظم الصحية والمشاركين، الناتجة عن التحول إلى نظم العلاج القصير للسل المقاوم للأدوية المتعددة.

الطريقة قمنا بالمقارنة بين تكاليف النظم الصحية والمشاركين في نظم العلاج الطويلة (20 إلى 22 شهرًا)، والقصيرة (9 إلى 11 شهرًا) للسل المقاوم للأدوية المتعددة في إثيوبيا وجنوب أفريقيا. تم جمع البيانات الخاصة بالتكلفة من المشاركين في المرحلة الثالثة من تجربة STREAM العشوائية الخاضعة للتحكم، وقدرنا تكاليف النظام الصحي باستخدام أساليب عملية تتدرج من القاع للقمة ومن القمة للقاع. تم إجراء تحليل فعال من حيث عن طريق حساب التكلفة التزايدية للنتائج غير المرغوب فيها التي تم تجنبها.

النتائج بلغت تكاليف الرعاية الصحية لكل مشارك في جنوب إفريقيا 8340.7 دولاراً أمريكياً (USD) بالنسبة للعلاج الطويل، و6618.0 دولاراً أمريكياً بالنسبة للعلاج القصير؛ في إثيوبيا، وكانت 6096.6 دولاراً أمريكياً و4552.3 دولاراً أمريكياً، على التوالي. كان أكبر مكون للتوفير هو تكاليف الأدوية في جنوب إفريقيا (67%؛ 1157.0 دولاراً أمريكياً من إجمالي 1722.8 دولاراً أمريكياً)، وتكاليف الدعم الاجتماعي في إثيوبيا (35%، 545.2 دولاراً أمريكياً من إجمالي 1544.3 دولاراً أمريكياً). في إثيوبيا، أعلن المشاركون في التجربة على النظام القصير عن انخفاض الإنفاق على الغذاء التكميلي (متوسط الانخفاض لكل المشارك: 225.5 دولاراً أمريكياً) وزيادة ساعات العمل (أي 667 ساعة إضافية عبر 132 أسبوعاً). إن احتمال أن يكون نظام العلاج القصير فعالاً من حيث التكلفة، كان أكبر من 95% عندما كانت القيمة المخصصة لتجنب النتائج غير المرغوبة فيها أقل من 19000 دولار أمريكي في إثيوبيا، وأقل من 14500 دولار أمريكي في جنوب أفريقيا.

الاستنتاج ارتبط نظام العلاج القصير لمرض السل المقاوم للأدوية بتخفيض ملموس في تكاليف النظام الصحي، وانخفاض العبء المالي على المشاركين.

## 摘要

目的 旨在调查因改用耐多药 (MDR) 结核病的短期治疗方案而引起卫生系统和参与者的费用变化。

**方法:**

我们比较了埃塞俄比亚与南非长期（20 至 22 个月）和短期（9 至 11 个月）耐多药结核病治疗方案对卫生系统和参与者产生的费用。费用数据是从 STREAM 第三期随机对照试验的参与者中收集的，并且我们采用自下而上和自上而下的方法估算了卫生系统的费用。通过计算防止不良疗效的人均增量费用进行了费用效益分析。

**结果:**

南非长期参与者的人均医疗护理费用为 8340.7 美元，短期参与者的人均医疗护理费用为 6618.0 美元；埃塞俄比亚长期参与者和短期参与者的人均医疗护理费用分别为 6096.6 美元和 4552.3 美元。南非最大的节省部分是药费（67％；总计 1722.8 美元中达 1157.0 美元），埃塞俄比亚最大的节省部分是社会支持费用（35％，总计 1544.3 美元中达 545.2 美元）。在埃塞俄比亚，短期方案的试验参与者报告补充营养食品的支出减少了（每位参与者平均减少：225.5 美元）并且增加了工作时间（即在 132 周中增加了 667 个小时）。当埃塞俄比亚防止不良疗效的价值低于 19,000 美元且南非防止不良疗效的价值低于 14,500 美元时，短期治疗具有费用效益的可能性大于 95％。

**结论:**

短期 MDR 治疗方案与卫生系统费用的大幅度降低以及参与者的经济负担减少有关**。**

## Резюме

**Цель:**

Изучение изменений в расходах для систем здравоохранения и участников в результате перехода на краткосрочную схему лечения туберкулеза со множественной лекарственной устойчивостью (МЛУ-ТБ).

**Методы:**

Авторы сравнили затраты систем здравоохранения и участников долгосрочных (от 20 до 22 месяцев) и краткосрочных (от 9 до 11 месяцев) схем лечения МЛУ-ТБ в Эфиопии и Южной Африке. Данные о затратах были получены от участников рандомизированного контролируемого клинического исследования фазы III STREAM. Авторы оценивали затраты системы здравоохранения, используя подходы «снизу вверх» и «сверху вниз». Оценка клинико-экономической эффективности выполнялась путем расчета дополнительных затрат на неблагоприятный исход, которого удалось избежать.

**Результаты:**

Расходы системы здравоохранения на одного участника в Южной Африке составляли 8340,7 долл. США для долгосрочной и 6618,0 долл. США для краткосрочной схемы лечения; в Эфиопии они составляли 6096,6 долл. США и 4552,3 долл. США соответственно. Самым крупным компонентом экономии были расходы на лекарственные препараты в Южной Африке (67%, 1157,0 долл. США от общей суммы 1722,8 долл. США) и расходы на социальную поддержку в Эфиопии (35%, 545,2 долл. США от общей суммы 1544,3 долл. США). В Эфиопии участники исследования по краткосрочной схеме лечения сообщали о более низких расходах на дополнительное питание (среднее сокращение на участника: 225,5 долл. США) и увеличении количества рабочих часов (то есть 667 дополнительных часов на протяжении 132 недель). Вероятность того, что краткосрочная схема лечения была более экономически рентабельной, превышала 95%, в то время как расходы на предотвращение неблагоприятного исхода в Эфиопии составили менее 19 000 долл. США, а в Южной Африке — менее 14 500 долл. США.

**Вывод:**

Краткосрочная схема лечения МЛУ-ТБ была связана со значительным сокращением расходов для системы здравоохранения и более низким финансовым бременем для участников.

## Introduction

Until recently, guidelines on multidrug-resistant (MDR) tuberculosis recommended a treatment period of 20 to 22 months,^1^ which has substantial costs for both patients and health services, particularly for hospitalization.^2^^–^^6^ A shortened treatment regimen of 9 to 11 months was tested in Bangladesh in 2010, with promising efficacy, and was subsequently implemented in several West African countries.^7^ However, no randomized controlled trials or economic evaluations have been performed. Given that health systems in many countries with a high MDR tuberculosis burden face resource constraints,^5^ there have been calls for more research on the economic impact of MDR tuberculosis. Moreover, global policy goals emphasize financial protection for patients and the elimination of catastrophic health-care costs.^8^

The results of the phase-III, noninferiority, randomized, controlled trial, STREAM, were published in 2019. They demonstrated that a short MDR tuberculosis regimen of 9 to 11 months had noninferior efficacy and comparable safety to the World Health Organization’s (WHO’s) approved standard regimen of 20 to 22 months (i.e. the long regimen).^9^ The trial collected data on the costs of each regimen for participants and health systems and on participants’ financial wellbeing.^10^^,^^11^ Our aim was to investigate the nature, magnitude and timing of the changes in costs for participants and health systems that result from switching to the short MDR tuberculosis regimen. As WHO’s treatment guidelines are undergoing rapid revision,^12^ we hope that our overall cost–effectiveness assessment and detailed cost analysis will help tuberculosis programme organizers to understand the potential costs and savings of transitioning to all-oral, short treatment regimens and to devise detailed plans for their implementation.

## Methods

The STREAM trial’s economic evaluation compared the health-system and participant costs of short and long regimens for treating MDR-TB in Ethiopia and South Africa. Before the trial, the median treatment duration was 20 months in Ethiopia and 22 months in South Africa. Trial participants were randomly assigned in a 2 : 1 ratio to the short or long regimen, with randomization stratified by trial site and the presence of human immunodeficiency virus infection.^11^ Data were collected at two sites in Ethiopia (i.e. St Peter’s Specialized Hospital and the Armauer Hansen Research Institute Hospital, both in Addis Ababa) and two in South Africa (i.e. Sizwe Tropical Diseases Hospital in Johannesburg and Doris Goodwin Hospital in Pietermaritzburg). Details of the methods are available elsewhere.^11^^,^^13^

We estimated health-system costs using a mix of bottom-up and top-down approaches.^14^^,^^15^ The costs of medications, inpatient stays and serious adverse events were calculated for individuals and the costs of laboratory tests, electrocardiography, staff time, consumables and social support were based on aggregate data collected during the trial. Where trial data were insufficiently detailed, we obtained supplementary information on typical care activities, such as tuberculosis drug use and the resources involved, by reviewing national and local guidelines and by interviewing clinical and managerial staff.^10^ We estimated costs using relevant unit costs for each country (available in the data repository).^13^

At some trial sites, participants were hospitalized from treatment initiation until they were smear negative. As accurate records of admission and discharge dates were unavailable, we used the time to sputum smear conversion as a proxy for the inpatient stay, allowing an additional 4 weeks for the result to be confirmed and communicated to clinicians. If a participant died within this period or before smear conversion, we assumed the hospital stay was the number of treatment days.

We also estimated the health-care resources required to manage serious adverse events because these events were the most costly.^16^ We estimated these costs for Ethiopia and based them on a sample of all serious adverse events associated with MDR tuberculosis or its treatment.^13^ Tests, examinations and care activities relating to the diagnosis and management of these events were identified by interviewing clinical staff and reviewing case notes.

Data on costs incurred by participants and on their socioeconomic status were collected at scheduled assessments between November 2012 and December 2017 in Ethiopia and between August 2014 and January 2018 in South Africa. The questionnaires used to assess participants’ costs were developed in English from the STOP-TB Partnership’s questionnaire,^17^ translated into local languages (i.e. Amharic, Zulu and Sesotho) and administered by the same staff who collected clinical data from trial participants. The questionnaires were administered 12 weeks after treatment randomization and every 12 weeks thereafter until the end of follow-up (i.e. 132 weeks). Information was collected on direct costs (e.g. food and transport) and indirect costs (e.g. lost income) incurred during the preceding 12 weeks. Participants were asked to estimate costs they would expect to face in routine care: for example, in South Africa, as free transport was provided for STREAM participants to attend clinic reviews, they were asked to estimate the usual cost of these trips. A separate questionnaire on participants’ socioeconomic characteristics was administered at randomization and then every 24 weeks. The number of participants at each site who provided data on direct costs, the cost of supplementary food and the number of hours worked is presented in Table 1.

**Table 1:** Participants providing information on direct costs of multidrug-resistant tuberculosis treatment, STREAM trial, Ethiopia and South Africa, 2012–2018

Information provided	No. of participants				
	Ethiopia			South Africa	
	St Peter’s Specialized Hospital (*n* = 68)	Armauer Hansen Research Institute Hospital (*n* = 51)		Doris Goodwin Hospital (*n* = 14)	Sizwe Tropical Diseases Hospital (*n* = 33)
**Direct costs of visiting health facility**	65	46		14	18
**Cost of supplementary food at treatment week:**					
12	35	20		9	2
24	50	25		12	5
36	48	26		13	6
48	53	22		13	2
60	57	30		0	0
72	59	36		0	0
84	54	38		11	3
96	48	35		4	7
108	50	42		2	2
120	49	41		6	2
132	61	39		14	0
**No. of working hours at treatment week:**					
24	56	26		11	6
48	56	30		13	9
72	53	37		13	6
96	39	38		5	0
120	47	41		6	0
132	60	38		0	5

STREAM: standard treatment regimen of antituberculosis drugs for patients with multidrug-resistant tuberculosis.

The study was approved by the International Union Against Tuberculosis and Lung Disease’s ethics advisory group, the South African Medical Research Council’s ethics committee, the Wits Health Consortium’s protocol review committee, the University of the Witwatersrand’s human research ethics committee, the University of Kwazulu–Natal’s biomedical research ethics committee, the St Peter TB Specialized Hospital’s ethical review committee and the Armauer Hansen Research Institute–All Africa Leprosy Rehabilitation and Training Hospital’s ethical review committee. All participants provided written informed consent. The trial registration number is ISRCTN78372190.

### Analysis

We estimated costs in 2017 United States dollars (US$) from the perspective of the health system and the participant separately.^18^ A trial-based perspective was adopted for estimating participants’ costs with a 132-week time horizon. Health-system costs were calculated for each participant who completed treatment – no follow-up costs were included because patients were not routinely followed up after the end of treatment. The cost of activities judged by the study’s clinical experts to have been solely for research (e.g. taking samples for pharmacokinetic studies) were excluded. 

A cost–effectiveness analysis was performed by calculating the incremental cost per unfavourable outcome avoided, which was the primary efficacy outcome of the STREAM trial. Unfavourable outcomes were defined as: (i) starting two or more drugs not in the allocated regimen; (ii) extending treatment beyond its scheduled end for any reason other than compensating for treatment not taken (up to a maximum of 8 weeks); (iii) death from any cause; (iv) a positive culture result when the patient was last seen; and (v) not seen at 76 weeks or later.^9^ Decision uncertainty was captured by conducting a probabilistic sensitivity analysis, which involved representing all uncertain parameters as probability distributions and propagating uncertainty using Monte Carlo simulations.^19^ The analysis was performed for Ethiopia and South Africa. Bootstrapping was used to account for uncertainty in parameters. We simulated 1000 estimates of mean costs and outcomes, which were used to construct 1000 simulated cost–effectiveness ratios. The results of the probabilistic sensitivity analysis are depicted in cost–effectiveness acceptability curves,^20^ which show the proportion of simulation results in which the short regimen was cost–effective. We assessed cost–effectiveness using a range of willingness-to-pay thresholds, which are payment thresholds that a decision-maker might assign to avoiding an unfavourable MDR tuberculosis outcome. We considered willingness-to-pay thresholds up to US$ 100 000 for both Ethiopia and South Africa.

#### Health-system costs

In Ethiopia, the cost of an inpatient stay was the sum of: (i) ward staff costs; (ii) inpatient overhead costs, which included hospital administration costs; and (iii) a fixed hotel cost, which included the cost of a bed, basic supplies and meals. For the two trial sites in Ethiopia, inpatient overhead costs were estimated using facility financial records. In South Africa, we based the estimates of basic inpatient unit costs on a published study.^3^ We judged this source to be the most appropriate as data were collected from a referral hospital similar in size to the two hospitals involved in the STREAM trial. A sensitivity analysis was carried out to explore how total costs would vary if unit costs from other studies were applied.^4^^,^^21^^,^^22^

#### Participant costs

We estimated the mean cost of a single health facility visit from participant-reported direct costs. The total cost incurred in routine practice was calculated by multiplying this mean by the number of visits expected during usual clinical management. For Ethiopia, missing values in participants’ responses were imputed using chained multiple imputation as the reference case.^23^ Two response categories included imputed values: (i) expenditure on supplementary food; and (ii) hours worked.^13^ Chained imputations could not be performed for South Africa because of a lack of data on both the imputed values and the variables included in the imputation model. All analyses of participants’ cost were performed in Stata v.15.1 (StataCorp LP., College Station, United States of America). Treatment of MDR tuberculosis involves an intensive phase (when five antibiotics are given daily, including an injectable) followed by a continuation phase (when at least four antibiotics are given orally). The intensive phase is costlier for patients because health facility visits are needed for the injections. There is also a greater risk of medication side-effects in this phase.

## Results

### Health-system costs

Table 2 gives details of the health-system costs for the short and long MDR tuberculosis treatment regimens. The cost was greater with the long than the short regimen: the total cost per participant in Ethiopia was US$ 6096.6 versus US$ 4552.3 (25% difference) for the two regimens, respectively, and in South Africa, US$ 8340.7 versus US$ 6618.0 (21% difference), respectively. Overall, 61% (US$ 944.3) of the reduction occurred in the continuation phase in Ethiopia, as did 85% (US$ 1461.3) in South Africa. In Ethiopia, the saving was primarily due to lower costs for social support (35%; US$ 545.2), laboratory tests (30%; US$ 456.9) and medications (20%; US$ 301.7), whereas in South Africa, the reduction was primarily due to lower medication (67%; US$ 1157.0) and staff costs (36%; US$ 619.1; Table 2). For the short regimen, the cost of cardiac monitoring per participant was US$ 149.5 in Ethiopia and US$ 150.9 in South Africa.

**Table 2:** Health-system costs of short and long multidrug-resistant tuberculosis treatment, STREAM trial, Ethiopia and South Africa, 2012–2018

Cost element, by country	Health-system costs in US$ per patient (% of country total)								Difference in health-system costs between long and short regimens in US$ per patient (% of country total)^b^		
	Long regimen^a^				Short regimen^a^						
	Intensive phase^c^	Continuation phase^c^	Total for two phases		Intensive phase^c^	Continuation phase^c^	Total for two phases		Intensive phase^c^	Continuation phase^c^	Total for two phases
**Ethiopia**											
Inpatient stay	2090.1 (50)	0.0 (0)	2090.1 (34)		2087.7 (59)	0.0 (0)	2087.7 (41)		2.4 (< 1)	0.0 (0)	2.4 (< 1)
Laboratory tests	381.0 (9)	469.6 (24)	850.6 (14)		197.2 (6)	196.5 (20)	393.7 (10)		183.8 (30)	273.1 (29)	456.9 (30)
Cardiac safety monitoring	0.0 (0)	0.0 (0)	0.0 (0)		79.8 (2)	69.8 (7)	149.6 (3)		−79.8 (−13)	−69.8 (−7)	−149.6 (−10)
Medication	1153.9 (28)	509.1 (27)	1663.0 (27)		969.5 (27)	391.8 (40)	1361.3 (33)		184.4 (32)	117.3 (12)	301.7 (20)
Staff	98.5 (2)	104.7 (5)	203.2 (4)		62.7 (2)	43.6 (4)	106.3 (3)		35.8 (6)	61.1 (7)	96.9 (6)
Social support	218.1 (5)	581.5 (30)	799.6 (13)		72.7 (2)	181.7 (19)	254.4 (6)		145.4 (24)	399.8 (42)	545.2 (35)
Consumables	163.2 (4)	244.8 (13)	408.0 (7)		81.6 (2)	102.0 (10)	183.6 (4)		81.6 (13)	142.8 (15)	224.4 (15)
Serious adverse events	60.5 (2)	21.6 (1)	82.1 (1)		14.1 (< 1)	1.6 (< 1)	15.7 (< 1)		46.4 (8)	20.0 (2)	66.4 (4)
Total	4165.3 (100)	1931.3 (100)	6096.6 (100)		3565.3 (100)	987.0 (100)	4552.3 (100)		600.0 (100)	944.3 (100)	1544.3 (100)
**South Africa**											
Inpatient stay	4284.5 (70)	0.0 (0)	4284.5 (51)		4480.2 (77)	0.0 (0)	4480.2 (68)		−195.7 (−74)	0.0 (0)	−195.7 (−11)
Laboratory tests	459.5 (8)	452.9 (20)	912.4 (11)		452.7 (8)	279.1 (35)	731.8 (11)		6.8 (3)	173.8 (12)	180.6 (10)
Cardiac safety monitoring	0.0 (0)	0.0 (0)	0.0 (0)		71.0 (1)	79.9 (10)	150.9 (2)		−71.0 (−27)	79.9 (−6)	−150.9 (−9)
Medication	621.0 (10)	969.9 (43)	1590.9 (19)		260.0 (4)	173.9 (22)	433.9 (6)		361.0 (138)	796.0 (54)	1157.0 (67)
Staff	643.6 (11)	692.5 (31)	1336.1 (16)		500.6 (9)	216.4 (28)	717.0 (11)		143.0 (55)	476.1 (33)	619.1 (36)
Social support^d^	0.0 (0)	0.0 (0)	0.0 (0)		0.0 (0)	0.0 (0)	0.0 (0)		0.0 (0)	0.0 (0)	0.0 (0)
Consumables	78.2 (1)	138.6 (6)	216.8 (3)		60.8 (1)	43.3 (5)	104.1 (2)		17.4 (7)	95.3 (7)	112.7 (7)
Total	6086.8 (100)	2253.9 (100)	8340.7 (100)		5825.3 (100)	792.7 (100)	6618.0 (100)		261.5 (100)	1461.3 (100)	1722.8 (100)

STREAM: standard treatment regimen of antituberculosis drugs for patients with multidrug-resistant tuberculosis; US$: United States dollar.

^a^ The long regimen lasted 20 to 22 months and the short regimen lasted 9 to 11 months.

^b^ Negative values indicate that costs were greater for the short than the long regimen.

^c^ In the intensive phase, five antibiotics are given daily (including an injectable); in the subsequent continuation phase, at least four antibiotics are given orally.

^d^ In South Africa, the cost of social support to the health system was zero because, unlike in Ethiopia, social support in South Africa was covered by donor funding.

Note: In South Africa, we were unable to estimate the cost of serious adverse events because care records were not available.

In Ethiopia, there was no substantial difference in the mean medication cost per participant between the regimens: it was US$ 1361.3 (95% confidence interval, CI: 1255.7 to 1465.8) for the short regimen and US$ 1663.0 (95% CI: 1536.4 to 1790.4) for the long regimen. In South Africa, however, there was a significant difference: the mean medication cost per participant was US$ 433.9 (95% CI: 385.4 to 481.1) for the short regimen and US$ 1590.9 (95% CI: 1283.5 to 1899.3) for the long regimen.

The largest expenditure category for both regimens was inpatient costs, even when the unit cost was varied in a sensitivity analysis.^13^ In Ethiopia, the mean inpatient stay was 9.62 weeks (95% CI: 9.01 to 10.24) for the short regimen and 9.64 weeks (95% CI: 8.74 to 10.52) for the long regimen. In South Africa, it was 9.43 weeks (95% CI: 8.30 to 10.56) for the short regimen and 9.02 weeks (95% CI: 7.51 to 10.52) for the long regimen. Consequently, changing to the short regimen had no meaningful implication for inpatient costs. The mean cost of a serious adverse event in Ethiopia was higher for the long (US$ 82.1; 95% CI: 46.0 to 118.2) than the short regimen (US$ 15.7; 95% CI: 1.2 to 30.2; Table 2). Although each episode was expensive to treat, the cost of serious adverse events did not substantially influence cost savings with the short regimen as few participants experienced them.

**Fig. 1:**
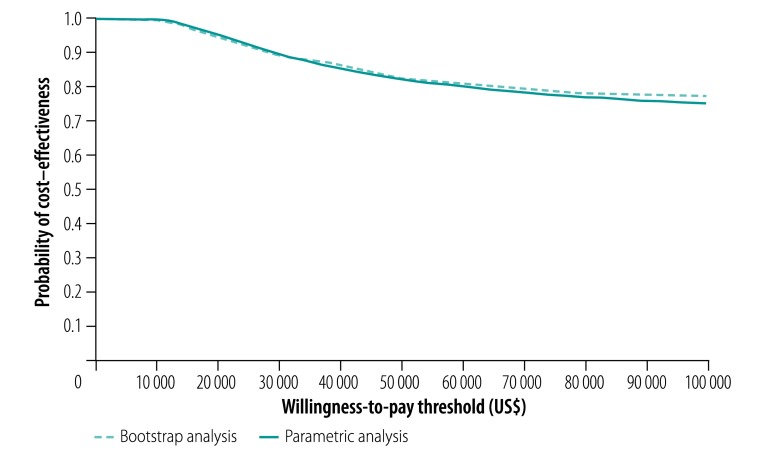
Probability that short multidrug-resistant tuberculosis treatment was more cost–effective than long treatment, by willingness to pay to avoid unfavourable outcomes, STREAM trial, Ethiopia, 2012–2017

STREAM: standard treatment regimen of antituberculosis drugs for patients with multidrug-resistant tuberculosis; US$: United States dollar.

Notes: Long treatment lasted 20 to 22 months and short treatment lasted 9 to 11 months. The willingness-to-pay threshold is the amount a decision-maker would pay to avoid an unfavourable outcome due to multidrug-resistant tuberculosis. For the parametric analysis, parameter values were simulated from distributions derived from the summary statistics of the observed data. For the bootstrap analysis, data were sampled with replacement values from the STREAM data set.

**Fig. 2:**
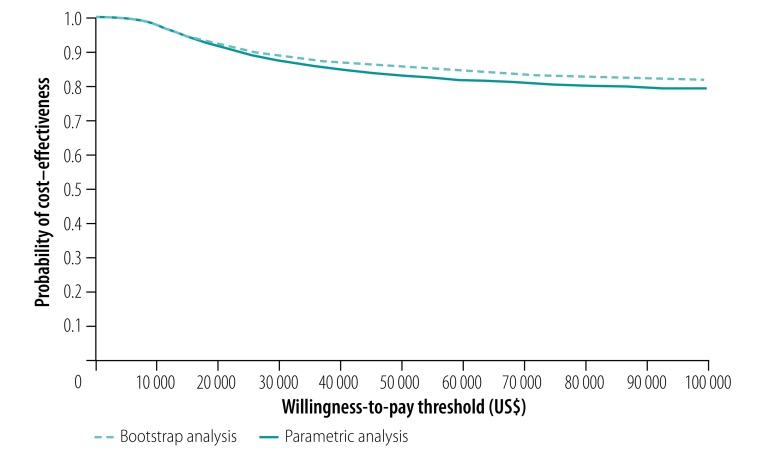
Probability that short multidrug-resistant tuberculosis treatment was more cost–effective than long treatment, by willingness to pay to avoid unfavourable outcomes, STREAM trial, South Africa, 2014–2018

STREAM: standard treatment regimen of antituberculosis drugs for patients with multidrug-resistant tuberculosis; US$: United States dollar.

Notes: Long treatment lasted 20 to 22 months and short treatment lasted 9 to 11 months. The willingness-to-pay threshold is the amount a decision-maker would pay to avoid an unfavourable outcome due to multidrug-resistant tuberculosis. For the parametric analysis, parameter values were simulated from distributions derived from the summary statistics of the observed data. For the bootstrap analysis, data were sampled with replacement values from the STREAM data set

Our probabilistic sensitivity analysis showed that the short regimen is highly likely to be cost–effective (Fig. 1 and Fig. 2). However, the probability it would be cost–effective declined as the value decision-makers placed on avoiding an unfavourable outcome increased: the probability was greater than 95% if that value were less than US$ 19 000 in Ethiopia and less than US$ 14 500 in South Africa. Even when the value was as high as US$ 100 000, the probability was still above 77% for both countries.

### Participant costs

Data for the participant-perspective analysis were available from 111 trial participants in Ethiopia and 14 in South Africa (Doris Goodwin Hospital). The mean cost per participant of a health facility visit was US$ 1.1 in Ethiopia (US$ 0.8 for transport and US$ 0.4 for food) and US$ 4.9 in South Africa (US$ 3.6 for transport and US$ 1.3 for food). In Ethiopia, as the short regimen was 11 months shorter than the long regimen, the cost saving per participant was US$ 12.5 over the treatment course. In South Africa, the difference was 13 months, giving a saving of US$ 64.0.

**Fig. 3:**
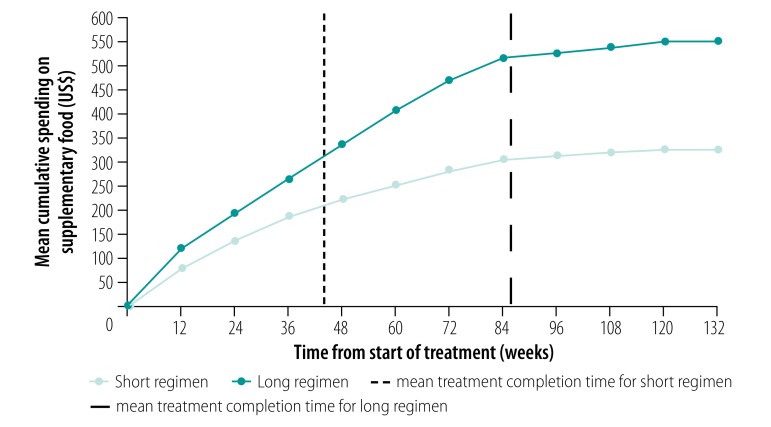
Participants’ cumulative spending on supplementary food, by length of multidrug-resistant tuberculosis treatment, STREAM trial, Ethiopia, 2012–2017

STREAM: standard treatment regimen of antituberculosis drugs for patients with multidrug-resistant tuberculosis; US$: United States dollar.

Notes: The long regimen lasted around 86 weeks and the short regimen lasted around 44 weeks. The dots represent data collection times. The nearest data collection time after completion of the short regimen was in week 48 and the nearest time after completion of the long regimen was in week 96.

In Ethiopia, 94% (104/111) of participants reported spending on supplementary food (e.g. meat, fruit and energy drinks). The cumulative mean per participant was US$ 549.1 (95% CI: 426.7 to 671.6) for the long regimen and US$ 323.6 (95% CI: 250.6 to 396.7) for the short regimen; the difference was US$ 225.5 (95% CI: 133.0 to 297.1; Fig. 3). The total direct costs per participant were US$ 575.4 for the long regimen and US$ 337.3 for the short regimen. Consequently, the total direct cost saving per participant with the short regimen was US$ 238.0, of which 95% related to reduced spending on supplementary food.^13^

**Fig. 4:**
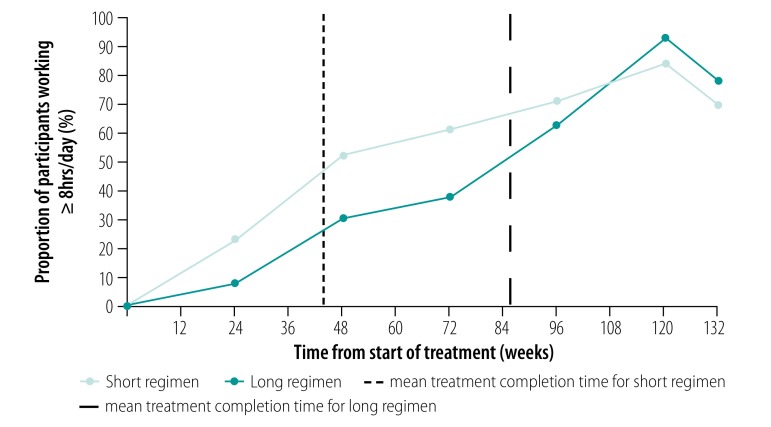
Proportion of participants working at least 8 hours per day, by length of multidrug-resistant tuberculosis treatment, STREAM trial, Ethiopia, 2012–2017

Participants in Ethiopia were unable or unwilling to provide estimates of their typical monthly income. However, many reported the number of hours they were able to work (Fig. 4). By 48 weeks after treatment initiation, an estimated 52% of participants on the short regimen were able to work at least 8 hours per day compared with 30% on the long regimen. Overall, the mean additional time worked per participant on the short regimen during the 132 weeks of treatment and follow-up was 667 hours (95% CI: 193 to 1127). This increase in productivity corresponded to a saving in indirect costs of US$ 175.7 per participant based on the reported incomes of MDR tuberculosis patients in Ethiopia.^24^ Consequently, the total cost saving per participant in Ethiopia was US$ 413.7 – 42% related to indirect costs and 58% related to direct costs. Insufficient data were available to estimate supplementary food expenditure and hours worked by participants in South Africa.^13^

STREAM: standard treatment regimen of antituberculosis drugs for patients with multidrug-resistant tuberculosis.

Notes: Work included schooling, housework and formal and informal work. The long regimen lasted around 86 weeks and the short regimen lasted around 44 weeks. All participants were hospitalized at randomization to treatment regimen. The percentages have been imputed as described in the methods section.

## Discussion

Using data from the phase-III, randomized, controlled STREAM trial, we found that the short regimen of MDR tuberculosis treatment led to substantial savings for both participants and the health-care system. Although this was intuitively expected, there were important, unexpected findings on the timing and drivers of these savings. We found that participant cost savings in Ethiopia were mainly due to lower expenditure on supplementary food and increased working hours; savings from fewer health facility visits were less important. The increase in working hours accrued largely between treatment weeks 16 and 32, when participants on the long regimen were receiving injectable drugs and those on the short regimen were not. Supplementary food expenditure diverged largely during weeks 48 to 84, when only those on the long regimen were still receiving treatment. These may be crucial benefits for MDR tuberculosis patients and their families given their typical socioeconomic situation. We estimated the mean cost to all trial participants in Ethiopia was 30 to 50% of their income,^24^ suggesting that a substantial number experienced catastrophic costs, though many fewer on the short regimen were affected.

Clinical and health-system factors, such as wages, prices and models of care, can also influence savings. For example, if inpatient care were maintained while patients receive injectable medications, switching to the short regimen (which involves four fewer weeks of injectable therapy) in South Africa would result in an additional saving of US$ 1958 per patient, thereby increasing the total saving to US$ 3681 per patient. We also estimated the effect on health-system costs in South Africa if outpatient care were the norm, which is increasingly common.^25^^,^^26^ Using published outpatient unit costs,^3^ the total health-system costs of the long and short regimens would be US$ 5600 and US$ 3415 per patient, respectively, both substantially less than for inpatient care (Table 2).

Cost savings also depended on the choice of antibiotics. In South Africa (but not Ethiopia), terizidone was used in the long regimen, whereas the medications used in the short regimen were heavily regulated, which gave substantial cost savings. Although participants on the short regimen needed cardiac monitoring due to the increased risk of a prolonged QTc interval, the cost of US$ 150 per participant was greatly outweighed by other savings.

Our study has limitations. Considerable data on participants’ responses were missing, particularly from South Africa where operational problems delayed data collection and reduced participants’ willingness to provide economic data. However, sensitivity analyses showed that these missing data had little impact on our findings.^13^ Moreover, the experience of trial participants was different from that of patients seen in routine practice, which could have influenced costs: the number of visits was different, and some support was provided (e.g. free or subsidized transport). Where possible, we adjusted our analysis to account for such differences. We did not include the costs or consequences of treatment failure, such as retreatment or increased morbidity and mortality. Short regimens could lead to an increased likelihood of retreatment or to more extensive drug resistance. However, no significant difference in unfavourable outcomes between the regimens was observed.

One limitation of our cost–effectiveness analysis is that we cannot definitively assert that the short regimen is cost–effective because the precise value placed on avoiding unfavourable outcomes was not available. Further research is needed to determine this value, which would involve estimating the costs and consequences of unfavourable outcomes. Nevertheless, the value would have to be hundreds of thousands of dollars before the short regimen becomes unlikely to be cost–effective.

In South Africa, we were unable to estimate the cost of serious adverse events because care records were not available. However, given the marginal difference in serious adverse events rates between regimens,^9^ it is unlikely they would have meaningfully changed our findings. Serious metabolic and nutritional disorders were more frequent in Ethiopia than in the trial overall (29%; 12/41, versus 9%; 12/141, respectively),^9^ probably because the injectable drug used was capreomycin, which has more metabolic side-effects than the kanamycin and amikacin used at other sites.

Despite these limitations, our study provides detailed comparative information on the health-system costs of treating MDR tuberculosis patients with different regimens. Furthermore, we found that the short regimen is associated with substantial savings for the health system, which are influenced by the local model of care. Nevertheless, the short regimen is highly likely to be cost–effective in other low- and middle-income countries. In addition, participants were able to return to work sooner, thereby helping safeguard the financial wellbeing of their households.

New evidence on the efficacy of short, all oral regimens for MDR tuberculosis will influence WHO’s considerations on whether to recommend a transition away from long regimens and the use of injectables.^12^ As we demonstrated, the economic implications of short regimens will vary considerably between countries. These variations are unlikely to change the overall economic case for shorter regimens, but they will be important for optimizing implementation. The switch to shorter regimens will involve stakeholders examining the local importance of the different cost categories we investigated in Ethiopia and South Africa and reflecting on their relevance for estimating budgets and developing implementation plans.
